# Feasibility of a virtual reality-based exercise intervention and low-cost motion tracking method for estimation of motor proficiency in youth with autism spectrum disorder

**DOI:** 10.1186/s12984-021-00978-1

**Published:** 2022-01-07

**Authors:** Darren R. Hocking, Adel Ardalan, Hisham M. Abu-Rayya, Hassan Farhat, Anna Andoni, Rhoshel Lenroot, Stan Kachnowski

**Affiliations:** 1 Developmental Neuromotor and Cognition Lab, School of Psychology and Public Health, La Trobe University, Melbourne, VIC Australia; 2 Zuckerman Mind Brain Behavior Institute, Columbia University, New York, NY USA; 3 School of Social Sciences and Humanities, Doha Institute for Graduate Studies, Doha, Qatar; 4 School of Psychology and Public Health, La Trobe University, Melbourne, VIC Australia; 5 HITLAB, Healthcare Innovation & Technology Lab, Columbia University, New York, NY USA; 6 Department of Psychiatry, University of New Mexico, Albuquerque, NM USA

**Keywords:** Kinect, Motion capture, Virtual reality, Video game, Artificial neural network, Motor skills, Technology-based intervention

## Abstract

**Background:**

Motor impairment is widely acknowledged as a core feature in children with autism spectrum disorder (ASD), which can affect adaptive behavior and increase severity of symptoms. Low-cost motion capture and virtual reality (VR) game technologies hold a great deal of promise for providing personalized approaches to motor intervention in ASD. The present study explored the feasibility, acceptability and potential efficacy of a custom-designed VR game-based intervention (GaitWayXR™) for improving gross motor skills in youth with ASD.

**Methods:**

Ten children and adolescents (10–17 years) completed six, 20-min VR-based motor training sessions over 2 weeks while whole-body movement was tracked with a low-cost motion capture system. We developed a methodology for using motion tracking data to quantify whole-body movement in terms of efficiency, synchrony and symmetry. We then studied the relationships of the above quantities with standardized measures of motor skill and cognitive flexibility.

**Results:**

Our results supported our presumption that the VR intervention is safe, with no adverse events and very few minor to moderate side-effects, while a large proportion of parents said they would use the VR game at home, the most prohibitive reasons for adopting the system for home therapy were cost and space. Although there was little evidence of any benefits of the GaitWayXR™ intervention in improving gross motor skills, we showed several positive correlations between the standardized measures of gross motor skills in ASD and our measures of efficiency, symmetry and synchrony from low-cost motion capture.

**Conclusions:**

These findings, though preliminary and limited by small sample size, suggest that low-cost motion capture of children with ASD is feasible with movement exercises in a VR-based game environment. Based on these preliminary findings, we recommend conducting larger-scale studies with methods for improving adherence to VR gaming interventions over longer periods.

## Background

Autism Spectrum Disorder (ASD) is a neurodevelopmental disorder with a prevalence of 1 in 54 children [1]. It has profound costs to both public health and individual families including lost productivity, which is projected to reach nearly half a trillion dollars over the next 5 years and to far exceed costs associated with diabetes and other conditions [2]. Although not included within the core ASD domains of restricted interests, repetitive behaviors and impaired social communication skills, motor impairments are pervasive in individuals with ASD. These impairments affect a variety of domains, including balance [3], movement planning [4], gait [5], and fine and gross motor coordination [6]. Motor difficulties in individuals with ASD appear to be present from birth [7] and persist across the lifespan [8, 9]. These motor challenges have been consistently found to be a precursor to later ASD symptoms and diagnosis [10, 11]. Additionally, motor difficulties, especially in postural stability, have been linked to autism symptom severity [12, 13]. Therefore, an important goal is to determine if we can improve motor skills in youth with ASD and develop sensitive outcome measures to track motor performance over time.

In order to determine which specific motor domains to target, several reviews of the motor literature have identified anomalous movement kinematics in youth with ASD [4, 14]. With regard to gross motor functioning, studies of postural control in ASD suggest abnormalities in the use of sensory feedback to maintain stability [3, 15, 16]. The extant studies have indicated a greater reliance on proprioceptive feedback alongside difficulties integrating visual information during balance [3, 17] and other upper extremity aiming tasks [4]. Further, it has been shown that individuals with ASD exhibit an atypically early plateau in their postural control development during adolescence with late improvements that typically do not reach adult levels [18]. Importantly, manual motor difficulties in ASD have been shown to be associated with adaptive behavior both concurrently [19] and longitudinally up to 12 years later [20]. These findings highlight the need to target balance and other motor skills through tailored interventions in youth with ASD to improve poor outcomes, and in such a way that is motivating and uniquely adapted to their needs.

An attractive type of intervention is to utilize active video gaming to help improve motor skills in youth with ASD. In recent years, several active video gaming platforms including Nintendo Wii Fit, Dance Dance Revolution, and Microsoft X-box Kinect, have been incorporated as training interventions to practice gross motor skills in autism and other developmental disabilities (see Hocking et al. [21] for a review). The commercially available off-shelf video games developed on these platforms have shown some effectiveness in increasing fitness levels (e.g. cardiorespiratory function, strength, speed, agility, and endurance) in children and adolescents with ASD [22]. However, a pilot feasibility study by Edwards et al. [23] found no evidence for sports active video games (Xbox Kinect) to improve object control skills in children with ASD and typically developing children. Thus, active video games alone may not provide adequate opportunities to influence the acquisition of motor skills in children with ASD. One particularly important limitation in using off-shelf video games is the lack of customizability and progression in level of difficulty required to target and personalize the acquisition of motor skills. That is, these off-shelf video games lack real-time adjustments in level of difficulty based on the user’s performance or commence with overly challenging tasks that exceed their individual ability level. Hence, there is a need to further develop customized video games for children with ASD, which capture real-time changes in motor performance to personalize the challenge at every level of the game using a closed-loop system.

To address these limitations, virtual reality (VR)-based interventions have been proposed as low-cost, scalable tools for motor training, notably because of their immersive and engaging aspects (i.e. VR 3D visualization and stereo sound can enhance the connection between user and the environment). Such interventions can highlight the role of movement variability in motor skill acquisition and enable transfer to complex real-world skills [24]. Several studies have highlighted the potential for VR gaming to improve gross motor performance in children with cerebral palsy [25], developmental delay [26], and Down syndrome [27]. To date, no research has investigated whether the use of VR game-based motor interventions are effective in improving gross motor skills in children with ASD. We developed GaitWayXR™ as a gaming platform that combines immersive virtual reality experiences with low-cost motion capture. Our game is designed to use a Microsoft Kinect camera and artificial neural networks for monitoring of real-time biomechanical changes and provide fine-tuning of the VR environment to update the challenge in real time. We attempted to overcome limitations of previous video game training platforms by creating a closed-loop system that (1) ensures the child is appropriately challenged by the training dosage, (2) the dynamic changes in the level of challenge is directly in response to the quality of the child’s movements and takes place in real-time, and (3) tracks fine-grained improvements in motor domains to update the challenge and provide opportunities to elicit the desired response.

To assess the effectiveness of our solution, the current feasibility pilot study combined a Microsoft Kinect camera and custom-designed VR game (GaitWayXR™) to create a 2-week motor training protocol for children with ASD. The Kinect camera was used to objectively and automatically quantify kinematic features and identify specific gross motor movements using machine learning methods. Here, we examined pre/post changes on standardized assessments of motor and cognitive skills, and baseline parent-reported autism symptom severity. The primary objective was to examine feasibility of using the GaitWayXR™ intervention to improve gross motor skills in children and adolescents with ASD. We measured feasibility by examining safety, tolerability, and usability with parent and child questionnaires and structured feedback. The secondary objective was to demonstrate a proof-of-concept for a method of quantifying dynamic whole-body movement from low-cost motion capture in children and adolescents with ASD. We developed a novel framework for objectively assessing motor skills from spatiotemporal features from motion tracking data during VR gameplay and correlated changes in these measures with changes in standardized assessments of motor skill and cognitive flexibility.

## Methods

### Participants

Participants were a convenience community sample of ten children with a previously confirmed diagnosis of ASD (based on previous medical records; no inclusion criteria based on ASD severity) aged 10–17 years (*M* = 14.10, *SD* = 2.6) including nine males and one female (see Table 1 for demographic characteristics). All participants were recruited through community autism clinics and inclusive settings in mainstream schools in Santa Monica and surrounding areas and written consents were obtained from parents/caregivers prior to participation in the study. The study obtained approval from the ASPIRE IRB in Santa Monica on 3/8/2019 (HITLAB-SS-2019). We started by assessing 21 participants, 14 of whom were determined to be eligible. Three participants were excluded due to lack of time/availability, behavioral problems or scheduling conflict, and one participant was withdrawn based on exclusion criteria after enrolling in the study. Participants were excluded if they were: (a) non-English speaking; (b) had a significant medical condition such as a major heart problem; (c) blindness, deafness (including seeing or hearing impaired), recent head, back or face injury; or (d) cerebral palsy, a diagnosis of Fragile X or Down Syndrome, or tuberous sclerosis. Participants were also excluded if they had epilepsy, sensitivity to flashing light or motion, unable to stand unassisted, against wearing the headset or have a psychiatric disorder stopping them participating in a VR-based environment.

**Table 1:** Demographic characteristics of the sample

Characteristic	*M*	*SD*	Range
Age	14.0	2.6	10–17
Male (n%*)*	9 (81.8%)		
Height (cm)	157.8	32.2	72.8–191
Weight	66.7	19.2	35.2–95.6
BMI	23.7	4.3	15.4–28.9
BOT-2 standard score	42.1	5.4	37–52
DCCS corrected score	90.1	16.5	59–112
SRS-2 Total Score (T-score)	71.2	10.4	54–85

### Measures

#### Safety, feasibility and usability measures

Any serious adverse events (e.g. seizure) and mild side effects that arise in VR (e.g. dizziness, headache, nausea, anxiety, disorientation) were recorded as measures of safety. In particular, we administered a Simulation Sickness Questionnaire (SSQ) at the end of the final session to evaluate the tolerability of the VR experience such as side effects including dizziness, blurred vision, fatigue, and heachache [28, 29]. The SSQ is rated on a four point scale (none, slight, moderate, severe). At the end of the final session, we also used a modified version of the System Usability Scale (SUS; [30]), a reliable measure to evaluate the usability of a system.

In addition to these two measures, we designed a post-study questionnaire that asked whether participants enjoyed playing the game, experienced any frustration from playing the game, would play the games outside the study, perceived any benefits in improving motor skills in the real world, and could see any prohibitive reasons in adopting the system for therapy purposes. They were also asked for areas of improvement and overall game structure, which were critical for direct stakeholder feedback and further refinements to the VR intervention. Refer to Table 3 for an example of the types of questions asked in the post-study questionnaire.

### Pre-post intervention measures

#### Bruininks-Oseretsky Test of Motor Proficiency, Second Edition (BOT-2 SF: [31])

The BOT-2 is a standardized assessment of motor proficiency that includes measures of stability, strength, mobility, coordination and object manipulation designed for individuals 4–21 years of age. The short-form BOT-2 test used in this study consists of 14 tasks that are clustered across 8 subtests of the complete BOT-2 and takes approximately 20–25 min to administer to the child. This assessment was conducted by a trained research assistant. The total score for the BOT-2 Short Form was calculated as the sum of the standard numerical scores on the different subtests, with a higher score representing greater motor proficiency.

#### Social Responsiveness Scale-2 (SRS-2: [32])

The SRS-2 is a 65-item parent/caregiver report designed to assess social communicative impairments that are characteristic of ASD. The measure consists of items that ask about reciprocal social behavior on a scale from “0” (never true) to “3” (almost always true). The SRS-2 is divided into five subscales: social awareness, social cognition, social communication, motor mannerisms and routines. Higher SRS-2 total score indicates greater severity of ASD symptoms. This measure was administred at pre-intervention only to chractarize overall severity of the child’s ASD symptoms.

#### NIH toolbox Dimensional Change Card Sort Test (DCCS: [33])

The DCCS is a measure of cognitive flexibility or set shifting. In this task, participants were asked to match a central target visual stimulus with 1 of 2 lateralized choice stimuli according to shape or color. Participants were presented with a pre-switch block consisting of five trials that were matched by the last attribute from the practice block, a postswitch block of five trials to be sorted by the other dimension, and a mixed block in which color or shape is relevant on the majority of trials with occasional, unpredictable shifts to the other dimension. Trials were presented in a pseudorandom fixed order, and the scores were based on pre- and post-switch blocks and the first 30 trials of the mixed block. Each vector score (maximum score of 10) incorporated both accuracy and reaction time (RT) for participants with a high level of accuracy (equal or greater than 80%) and accuracy only for those who did not obtain this criterion. Higher DCCS scores correspond to better cognitive flexibility.

### Procedures

#### Intake sessions

This pilot study was conducted in a purpose-built VR play area in offices in Santa Monica and participants were initially invited to a telephone screening to determine eligibility and then invited to visit the VR laboratory if they met eligibility requirements. The intake session included parent reports of autism symptom severity, and the DCCS and BOT-2 assessments. Participants were then immersed in a comprehensive pre-training session in VR using a character-driven avatar (Dr. Knows) to detail the backstory and demonstrate the core movements (calibration trials) that the child was required to copy while being tracked on the Kinect camera. These movements were part of a calibration phase that determined the level of difficulty for the starting trials of the training protocol.

#### In-lab Kinect camera set-up

**Fig. 1:**
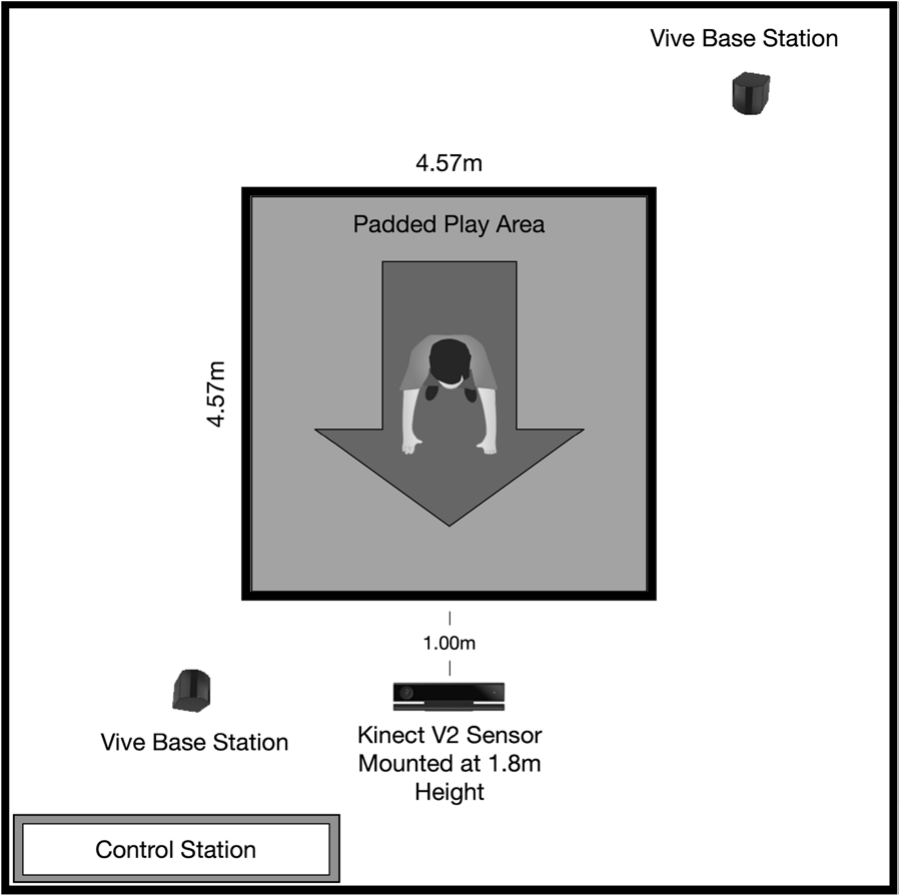
Schematic of in-lab VR-based videogame intervention setup

During the intake sessions, in-lab training sessions were conducted to customize the level of difficulty to each individual’s starting ability on each of the core movements. Figure 1 illustrates our in-lab setup. The game software integrated a Microsoft Kinect V2 camera with the customized GaitWayXR™ platform, with the Kinect camera tracking movements that were recognized by a fully customized machine learning (ML) model. Participants were positioned in the center of a testing area and fitted with a wireless HTC Vive Pro headset, with tripods holding the headset’s base stations positioned at each corner of the testing area at a fixed height. The Kinect camera was positioned directly in front and approximately 1 m away from the testing area (see Fig. 1).

#### Intervention period

**Fig. 2:**
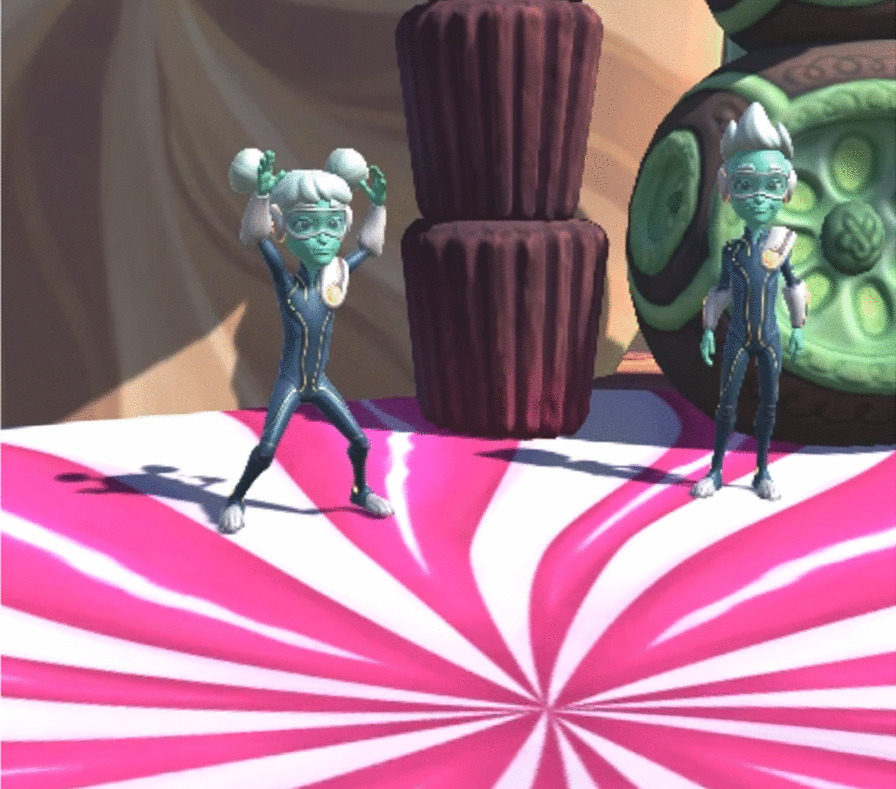
Screenshot of prescribed movements (jumping jacks) performed during the CandyDance game demonstrated by an animated character in the in-lab VR-based play session

Participants were asked to complete the same six, 20-min VR-based play sessions over two weeks with individual adjustments to level of difficulty from session 1 to 6. The VR immersion involves engaging in two types of games and progressing through levels in a 4.57 × 4.57 m well lit, padded play area. The dance game (Candy Dance) included 5 levels of dance moves with increasing complexity where participants were asked to perform movements previously recommended by an occupational therapist (OT) and demonstrated by an animated character (Princess Carmella). The following movements were performed (see Fig. 2): jumping jacks were performed from a standing position by jumping to a position with legs spread and arms raised and then back to the original position; an idle pose was arms to the side with little to no movement, a step to the left was taking one full step to the left from the center of the screen, step right is taking one step right from the screen, jump forward was jumping forward a minimum distance of 30 cm, same for back/left/right. The arena and dances were always choreographed in a way that forced the user back to the center to avoid any injuries. We considered 3–4 strides as a walk, and on average 10 of each move was collected in each session. The first level involved slow and easy movements and increased to more difficult combinations of movements at each subsequent level based on an adaptive staircase algorithm that generated 75–85% correct performance. The frequency of more difficult motions increased based on a real-time analysis of number and quality of correct movements captured in real-time by the Kinect camera; this ensured that each child was optimally engaged, and the challenge was updated continuously based on their individual performance.

#### Post-intervention

Following the intake and intervention sessions, the BOT-2 and DCCS were completed again. Children and parents were also asked to complete questionnaires at this final visit, providing feedback on system usability, tolerability and simulator sickness, as well as overall impressions of the game and areas for improvement.

### Data acquisition and pre-processing

The x, y and z coordinates from the twenty-five joints from the skeletal model of the Kinect V2 system were recorded concurrently with the GaitWayXR™ game. These coordinates were time stamped and stored at a sampling rate of 30 Hz. Data from the Kinect was filtered using a 12 Hz low-pass filter to reduce the influence of noise during tracking. In order to reduce the variability caused by different positioning of participants in front of the Kinect camera, the joint coordinates were shifted such that the pelvic joint was positioned at the origin (a processing step to ensure the data was agnostic to the participants’ position within the camera scene). The data was stored in a secure local (no access to the internet) password protected database with no identifiable information and unique participant ID. Other participant information that was stored included number of sessions, baseline measures at start of session, and the maximum level difficulty achieved during the VR experiences.

### Data segmentation

The data segmentation step took as input individual video recordings of participants while playing the game, and for each video, it generated a collection of movements (e.g. jump left, step forward and jumping jack; see below for the complete list), each of which consisted of a sequence of frames in the video. To do so, a realtime sliding window of frames captured by the Kinect camera was maintained and updated. Once 60 frames were collected, they were sent to a neural network to recognize the current action. The action recognition results were updated every 15 frames of novel data (0.5 s). Once a frame had been identified, it was sent to the relevant analysis portion of the game. To correct for errors, we manually checked that all of these motions were identified at the correct time for each dance move in CandyDance.

We used an artificial recurrent neural network for action recognition which took a 60 (frames) × 52 (joint coordinates) dimensional representation of the motion data as input for action recognition and generated one of 10 output labels for the current data window. The labels were jumping jack, jump forward, jump backward, jump right, jump left, walk, step forward, step back, touch nose, and idle.

The action recognition neural network consisted of an input layer of 3129 (= 60 × 52) dimensions, three hidden long short-term memory (LSTM) [34] layers of 20 nodes each, and a softmax output layer of 10 dimensions. The softmax function applies a normalized exponential transformation to the outputs’ linear combinations, converting them to a discrete probability assignment over the 10 possible labels. It was trained from a small dataset collected and augmented by flipping the data on the Y axis to double the dataset, then by time warping the motions to various speeds (+ 20% increase/decrease) at 5% increments. A total of 2000 raw motions were collected, 4000 after first step augmentation, and 24,000 after second step augmentation (6 * 5% increments). The training set consisted of 10 movements from a larger range of OT prescribed movements collected from ten participants involving 6000 + samples and 360,000 frames of motion data. To attain generalization, we used a testing set that comprised 20% of the initial dataset. The model was trained until no improvement was seen on the test set accuracy for 50 epochs, and then the model with the highest validation accuracy was exported and used in the final program.

The VR games were programmed and deployed using Unity as the game engine (Unity 2018.3). The software integrated the Kinect camera to perform pose estimation and track fine-tuned biomechanical changes during video gameplay. The action recognition network was deployed in Unity using TensorFlow Sharp (Tensorflow 1.13) as a wrapper to construct the recurrent neural network. The neural networks were created and trained in Keras, a high-level wrapper for TensorFlow (Keras 2.2.4; Google, 2015).

### Data segmentation cleaning

To study the participants’ motor profiles in detail, we focused on one particular movement, i.e. jumping jacks. This was the most sophisticated movement among the ones performed by the participants and we selected this movement for our analysis because it enabled us to quantify the differences in motor skill of our participants. Since we needed these movements and their start/finish times to be highly accurate, we manually checked and adjusted the results of the data segmentation step as follows. We went through all of the movements labelled as a jumping jack by the action recognition network and for each of those movements, we checked the start/finish frames to make sure that they were accurate. If not, we manually corrected the start and/or finish frames to reflect the correct values.

For each jumping jack (JJ) movement, we then cleaned the data by first denoising the time series corresponding to the dimensions of each extracted joint coordinates (e.g. x dimension of left shoulder). This step helped with removing the high-frequency (HF) parts of the collected signals which were not physically possible for the participants to perform and were a result of the noisy extraction process. To clean the signals, we used the 2^nd^ order zero-lag Butterworth filter with cut-off frequency of 3 Hz to remove the high-frequency components of the movement signals.

**Fig. 3:**
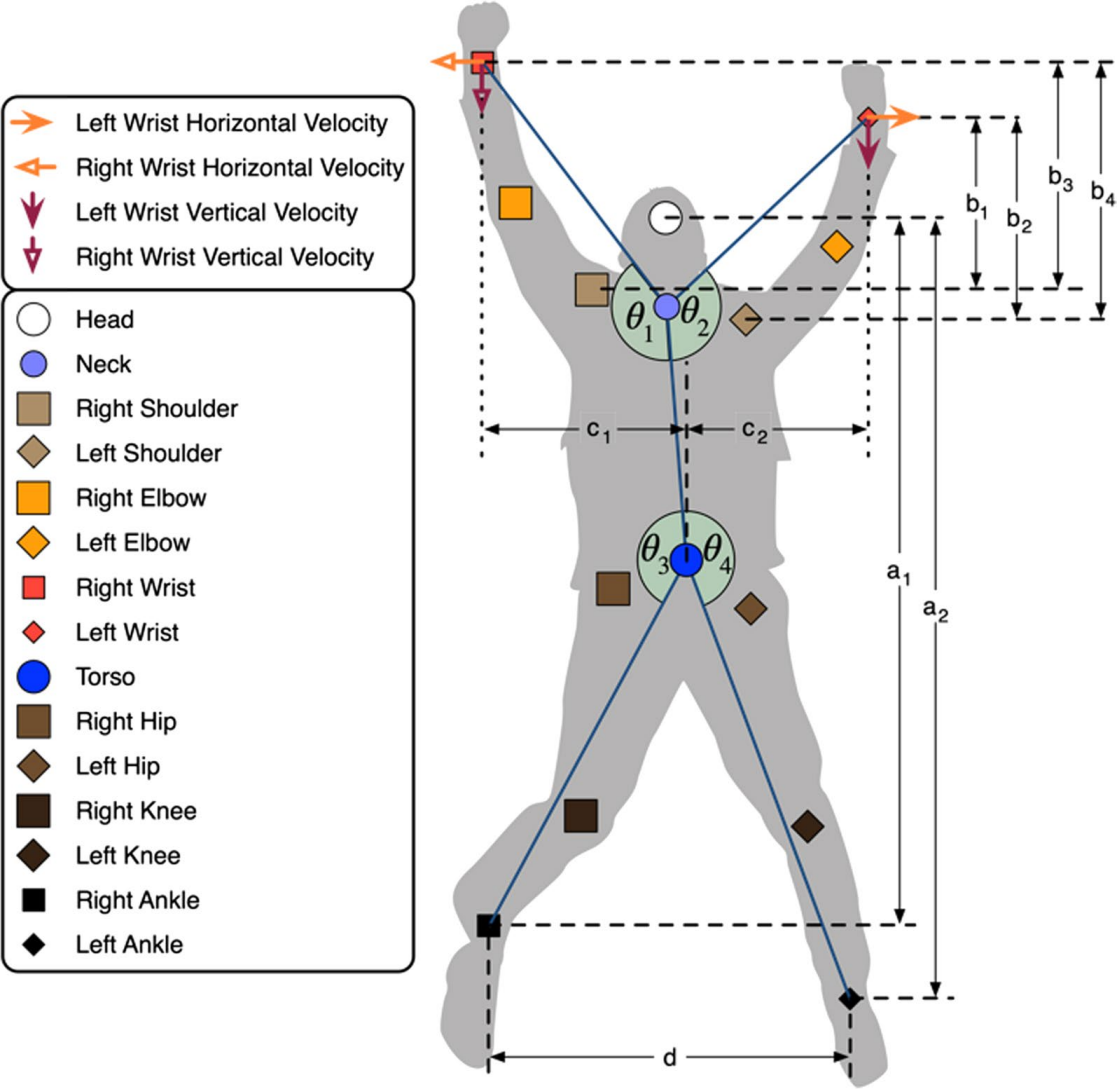
Quantities used to calculate various measures on jumping jack movements

We then quantified each jumping jack (JJ) movement performance by calculating various measures in three main categories: (1) efficiency measures, evaluating how efficiently a participant executes a JJ (i.e. the limits of how far their distal limbs can go), (2) synchrony measures, evaluating how coordinated a participant is while performing a JJ (i.e. quantify how different limbic angles are synchronized together), and (3) symmetry measures, evaluating how symmetrical the participant performs a JJ (i.e. evaluate how symmetrical the positions and velocities of their limbs are). Table 2 shows the measures in each category along with their description and formulae, and Fig. 3 illustrates some of the quantities used to calculate these measures.

**Table 2:** Details of various measures used to quantify jumping jack movements

Category	Name	Description	Formula
Efficiency	*K*_*1*_	Highest wrist position normalized by height: Maximum difference wrists and shoulders heights, divided by height	$D_{w/h} = E_{t} \left[ {\frac{{\left( {b_{1} , \ldots ,b_{4} } \right) }}{{\left( {a_{1} ,a_{2} } \right) }}} \right]$
	*K*_*2*_	Widest leg split normalized by height: Widest distance between the ankles, divided by height	$D_{b/h} = E_{t} \left[ {\left( {\frac{d}{{\left( {a_{1} ,a_{2} } \right) }}} \right) } \right]$
Synchrony	*H*_*1*_	Dominant frequency variance: Variance of dominant frequencies of articulated figure angles ${\theta }_{1},\dots ,{\theta }_{4}$ where dominant frequency ${f}_{i}^{max}$ is the frequency on the fast Fourier transform spectrum of ${\theta }_{i}$ with highest magnitude	$\sigma_{{f^{max} }}^{2} = \frac{1}{3}\sum\nolimits_{i = 1}^{4} {\left( {f_{i}^{max} - \mu_{{f^{max} }} } \right)^{2} }$ $\mu_{{f^{max} }} = \frac{1}{4}\sum\nolimits_{j = 1}^{4} {f_{j}^{max} }$
	*H*_*2*_	Mean absolute relative phase: Average difference in absolute value of instantaneous phase angles (PAs) of two signals	$MARP_{{s_{1} ,s_{2} }} = E_{t} \left[ {\left| {PA_{{s_{1} }} - PA_{{s_{2} }} } \right|} \right]$ $PA_{s} = \left( {\frac{{s^{\prime}}}{s}} \right)$
	*H*_*3*_	Continuous relative phase standard deviation: Standard deviation of continuous relative phase of two signals	$CRPSD_{{s_{1} ,s_{2} }} = \sqrt {var_{t} \left[ {PA_{{s_{1} }} - PA_{{s_{2} }} } \right]}$
	*H*_*4*_	Average of hand stop differences: Average difference in absolute value of time instants at which left and right arms stop moving, e.g. at the apex of JJ	$A_{w} = \left[ {\frac{1}{{\left( {n_{R} ,n_{L} } \right) }}\sum\nolimits_{k = 1}^{{\left( {n_{R} ,n_{L} } \right) }} {\left| {T_{i + k}^{{w_{R} }} - T_{j + k}^{{w_{L} }} } \right|} } \right]$
	*H*_*5*_	Average of leg stop differences: Average difference in absolute value of time instants at which left and right legs stop moving, e.g. at the apex of JJ	$A_{l} = \left[ {\frac{1}{{\left( {m_{R} ,m_{L} } \right) }}\sum\nolimits_{k = 1}^{{\left( {m_{R} ,m_{L} } \right) }} {\left| {T_{i + k}^{{l_{R} }} - T_{j + k}^{{l_{L} }} } \right|} } \right]$
Symmetry	*M*_*1*_	Average hands bilateral symmetry: Average difference in absolute value of horizontal distance between the hands	$\mu_{{X^{w} }} = E_{t} \left[ {\left| {c_{1} - c_{2} } \right|} \right]$
	*M*_*2*_	Standard deviation of hands bilateral symmetry: Standard deviation of difference in absolute value of horizontal distance between the hands	$\sigma_{{X^{w} }} = var_{t} \left[ {\left| {c_{1} - c_{2} } \right|} \right]$
	*M*_*3*_	Horizontal hand velocities bilateral symmetry: Average difference in absolute value of horizontal velocities of left and right hands	$\mu_{{V_{ \to }^{w} }} = E_{t} \left[ {\left| {V_{ \to }^{{w_{R} }} - V_{ \to }^{{w_{L} }} } \right|} \right]$
	*M*_*4*_	Vertical hand velocities bilateral symmetry: Average difference in absolute value of vertical velocities of left and right hands	$\mu_{{V_{ \downarrow }^{w} }} = E_{t} \left[ {\left| {V_{ \downarrow }^{{w_{R} }} - V_{ \downarrow }^{{w_{L} }} } \right|} \right]$

### Statistical analyses

To address the first study objective, we analyzed participants’ data on the feasibility, tolerability and usability scores, and their perceptions of training benefit and enjoyment using a series of descriptive and frequency statistics, as well as the changes they experienced on the BOT-2 and DCCS measures, using Wilcoxon Signed Ranks test for paired samples. To address the second study objective, we used Spearman’s rho correlations to examine relationships between changes in the efficiency, synchrony, and symmetry scores that occurred between session 1 (start) and session 6 (end) of the intervention (using the mean scores) with pre-post test changes on the BOT-2 and DCCS measures. Non-parametric tests (Wilcoxon and Spearman’s rho) were employed in the analyses due to the small sample.

## Results

### Feasibility, tolerability and usability

**Fig. 4:**
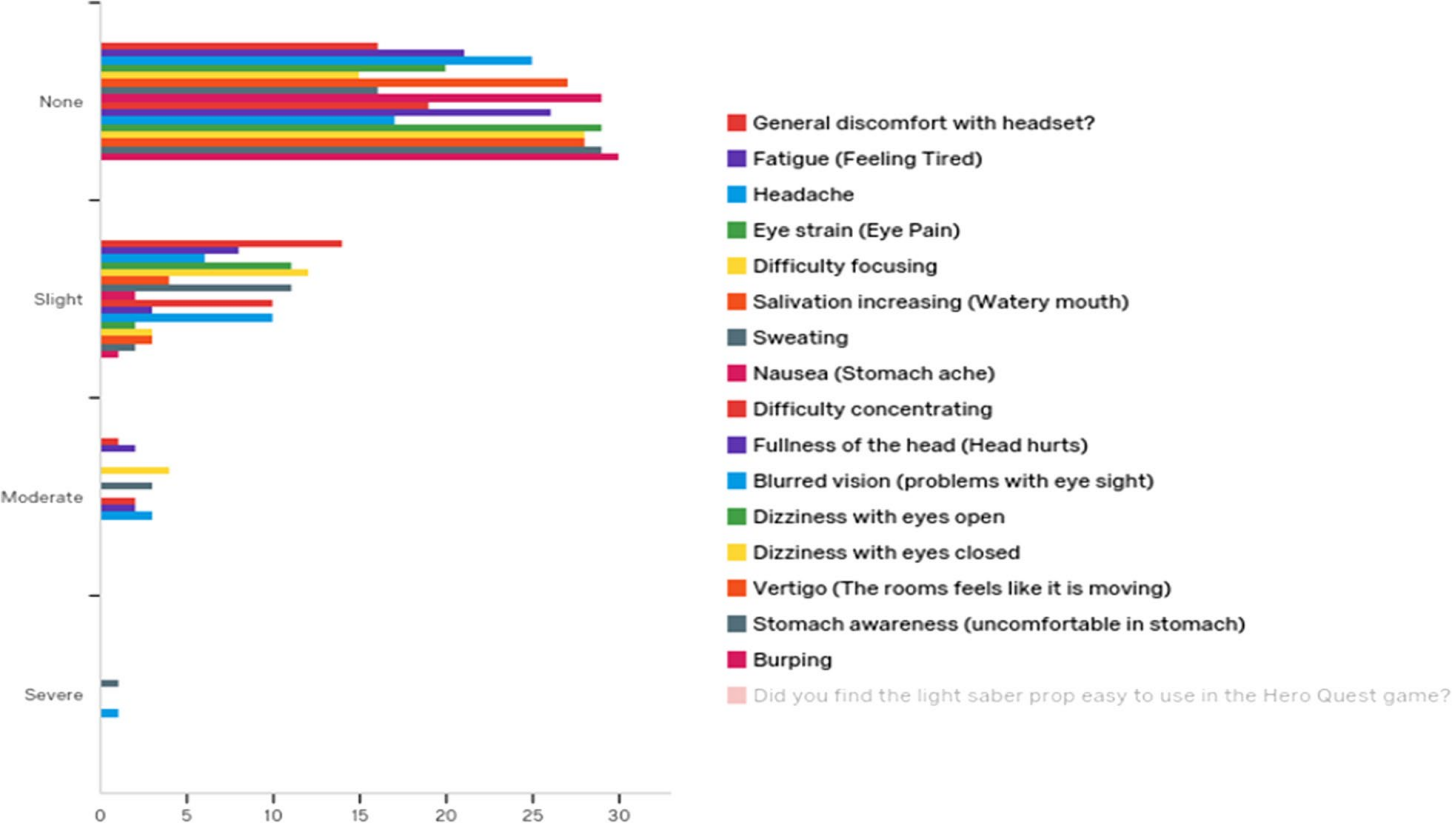
Frequency of side effects of simulation based on severity (none, slight, moderate, severe) and type of symptom using the Simulation Sickness Questionnaire (SSQ)

There were no serious adverse events recorded, although mild to moderate side effects arising from VR were reported. The six 20 min sessions of VR was feasible, such that all participants beginning the VR session were able to complete the entire training sessions. The SUS scores averaged 69.7 (*SD* = 11.1), which was considered average with room for improvement in usability based on population norms [35]. The vast majority of participants experienced no or only slight side effects over the course of the six VR sessions. There was increased frequency of moderate side effects for general discomfort, fatigue, difficulty focusing, sweating, nausea, fullness of the head, blurred vision; and some severe side effects were reported for eye strain and blurred vision. Overall, there were mild to moderate side effects reported by participants across all of the sessions based on the SSQ scores (see Fig. 4).

### Perceptions of training benefit and enjoyment

The ratings by the participants showed that the vast majority enjoyed playing the games and would continue to play the game outside of the study context (70%), although a large percentage of parents were unsure if the game would improve motor skills in the real world (70%) (see Table 3). The vast majority of participants with ASD found the tutorial and instructions easy to understand (90%). Parent ratings showed that children enjoyed playing the game (*Mean* = 7.14). The most prohibitive reasons for adopting the system for home therapy identified by parents were cost (50%) and space (30%) requirements.

**Table 3:** Feedback questionnaire from children and parents on perceptions of training benefit and enjoyment

Feedback Questionnaire: child rating	
Was the tutorial in the GaitWay Village easy to understand?	
Yes	90%
No	10%
Were the instructions easy to understand?	
Yes	90%
No	10%
Do you want to play again at home?	
Yes	70%
No	30%
No	10%

Feedback Questionnaire: parent rating	
How much do you think your child enjoyed playing the games in the virtual reality headset on a scale of 1–10?	M = 7.14
Did your child exhibit any frustration with the game?	
Yes	40.00%
No	60.00%
Did they talk about the game after the session?	
Yes	70%
No	30%
Do you think that playing the game helped your child improve motor skills in the real world?	
Yes	20%
No	10%
Unsure	70%
How would you rate the time your child spent playing the study application?	
Very worthwhile	50.00%
Somewhat worthwhile	50.00%
Not worthwhile	0.00
Would you use this at home?	
Yes	60%
No	40%
What would be the most prohibitive to adopt this system for therapy purposes for your child?	
Cost	50%
Space requirements	30%
Game addiction potential	10%
Time	10%
Would you limit the time your child spends playing virtual reality games?	
Yes	90%
No	10%

### Pre- and post-intervention changes

Wilcoxon Signed Ranks test indicated non-statistically significant changes from pre- to post-intervention in the average values of both the BOT-2 (Pre-*Mean* = 42.11, *SD* = 5.44; Post-*Mean* = 43.33; *SD* = 4.69; *z* = − 1.68, *p* = 0.093) and DCCS measures (Pre-*Mean* = 90.13, *SD* = 16.53; Post-*Mean* = 90.22; *SD* = 13.59; *z* = − 0.51, *p* = 0.61).

### Efficiency, synchrony, and symmetry

Spearman’s rho (*r*_(s)_) correlations between pre and post scores on the BOT-2 gross motor skills and DCCS cognitive flexibility measures were, repectively, 0.87 (*p* = 0.002) and 0.75 (*p* = 0.03). Table 4 shows Spearman’s rho (*r*_(s)_) correlations between changes that occurred in JJ movements performed from the first to the last completed sessions for all the participants and pre-post changes in the BOT-2 and DCCS standardized clinical assessment scores. We observed statistically significantly positive correlations between at least one measure in each of the efficiency, synchrony and symmetry categories with pre-post changes in BOT-2 gross motor skills. The significant correlation coefficients were moderate to strong, ranging from 0.47 to 0.82. About 22% to 67% of the variance of the improved BOT-2 gross motor proficiency scores from session 1 to session 6 was accounted for by positive changes in efficiency (*K2*), synchrony (*H2*, *H3*, *H5*) and symmetry (*M1*, *M2*) scores from session 1 to session 6. Changes in the JJ movements through the intervention were not correlated with pre-post DCCS cognitive flexibility scores.

**Table 4:** Spearman’s rho correlations (*r*_(s)_) between Session 1—Session 6 changes in quantitative measures extracted from motion tracking and pre-post BOT-2 and DCCS scores

	Efficiency		Synchrony					Symmetry			
	K_1_	K_2_	H_1_	H_2_	H_3_	H_4_	H_5_	M_1_	M_2_	M_3_	M_4_
BOT-2	0.36 (0.27)	**0.82** (0.02)	0.36 (0.25)	**0.82** (0.03)	**0.67** (0.035)	0.10 (0.42)	**0.67** (0.039)	**0.57** (0.033)	**0.47** (0.04)	0.10 (0.44)	0.40 (0.25)
DCCS	0.04 (0.6)	0.08 (0.7)	0.04 (0.7)	0.20 (0.3)	0.20 (0.4)	0.08 (0.6)	0.20 (0.5)	0.01 (0.8)	0.40 (0.3)	0.08 (0.6)	0.01 (0.8)

*BOT-2* Bruininks-Oseretsky Test of Motor Proficiency, Second Edition, Gross Motor Proficiency; *DCCS* NIH toolbox Dimensional Change Card Sort Test; K1-K2, H1-H5, and M1-M4 are described in detail in Table 2. Boldfaced correlations are significant (*p* < .05). p-values in parentheses

## Discussion

The present study provides the first pilot feasibility data on a novel intervention setup targeting movement skills in children with ASD. This setup consists of a custom-built virtual reality game (GaitWayXR™) combined with low-cost motion capture technology (Kinect V2 camera) to adapt the gameplay (i.e. difficulty level) in real time. The GaitWayXR™ platform involved balance and whole-body movements over six sessions using an immersive VR game and the commercially-available Kinect V2 camera provided quantitative real-time feedback to the game engine in order to adjust the gameplay challenge. Our findings, though preliminary and limited by small sample size, suggest that the novel VR intervention did not improve gross motor proficiency and cognitive flexibility of ASD children. However, our findings indicated that it is feasible to combine movement exercises and low-cost motion capture for motor intervention in children with ASD. Our Kinect software tool to quantify movement performance (i.e. jumping jacks) showed moderate to strong positive correlations between changes in certain efficiency (*K2*), synchrony (*H2*, *H3*, *H5*) and symmetry (*M1*, *M2*) indicators from session 1 to session 6 and changes in standardized pre-post measures of motor clinical assessment in ASD. Parents and participants rated the GaitWayXR™ protocol as generally acceptable with no adverse events and mild to moderate side-effects. While a large proportion of parents said they would use the intervention at home, the most prohibitive reasons for adopting the system for home therapy were cost and space. Below, we discuss these findings and provide recommendations about how the VR game can be improved to encourage repetitive practice and adherence over a longer period of time required to improve gross motor performance in youth with ASD.

Our finding that all of the participants completed the multiple VR session training protocol demonstrates the feasibility of completing a short, two-week VR game in youth with ASD. Acceptability results show that the majority of participants found the instructions easy to understand and a large proportion felt the VR game was easy to understand and something they would continue to play at home. However, among the proportion of parents who would not adopt the VR system for therapy purposes for their child at home, cost and space requirements stood out as prohibitive factors. One potential reason to explain this might relate to the use of a tethered PC-based VR headset in the current study, which has limitations in terms of lack of portability and high cost. However, there have been significant advances in VR systems since the time period of this study [36], and therefore one method of improving adoption and acceptability might be to utilize these standalone VR headsets (e.g. Oculus Quest) for home-based exercises in individuals with ASD. Another important consideration is greater involvement of key stakeholders (e.g. children, clinicians, service providers etc.) during the development of any future VR game intervention using a co-design process to ensure high acceptability and that the intervention meets their unique needs.

With regard to preliminary efficacy, our results revealed that the VR game-based motor intervention did not show statistically significant changes on the gross motor proficiency and cognitive flexibility measures between the pre- and post-intervention assessment. This is perhaps not surprising given the preliminary nature of our study, small sample size, and the short duration and dosage of 3 × 20 min VR sessions/week over a period of two weeks, which is likely to have weakened any intervention effect. Notwithstanding the limitation of small sample size, our findings from a non-invasive motion capture approach suggest that it is feasible to sensitively measure dynamic whole-body movement based on close relationships between our feature-based motion tracking measures and standardized motor assessments during VR game play in youth with ASD.

Our non-invasive approach to categorize a specific jumping jack movement captured by a Kinect camera showed that it is feasible to measure complex whole-body movement using low-cost motion capture in youth with ASD. During the VR gaming intervention, we distinguished movements using three categories of measures (efficiency, synchrony and symmetry) and our results suggest that changes in synchrony over the intervention course was the most sensitive, to individual differences in gross motor proficiency as measured by a pre-post BOT-2 motor assessment. In particular, improvement in three synchrony indicators (*H2*, *H3*, *H5*) out of five, compared to two symmetry indicators (*M1*, *M2*) out of four and one efficiency indicator (*K2*) out of two were significantly correlated with improvement in gross motor proficiency in the ASD group. The positive relationship between higher degree of efficiency, synchrony, more symmetrical movements, and improved motor proficiency in our VR intervention aligns with a previous study examining the effects of a biofeedback-based balance training intervention on improving balance in youth with ASD [37], with a clear demonstration that ML classifications of whole-body movement tracking (using a Kinect camera) were correlated with balance during one-footed standing [38]. Our measure of synchrony depends on the integration of visual input with motor output involving simultaneous occurrence of action sequences with respect to both timing and speed [39]. Reduced visual-motor synchrony has been previously reported in children with ASD [40], and is associated with more severe autistic traits [41]. On the other hand, efficiency is the ability to manipulate body position, to maintain balance and execute intentional movement relative to energy expended [42]. Thus, our feature-based algorithms for motion tracking improvements showed a close correspondence with standardized assessments of motor proficiency in youth with ASD.

These findings, which need to be confirmed in studies with a larger sample size, suggest that our quantitative measures could be useful in tracking improvements in core motor symptoms in response to VR-based gaming interventions in ASD.

Given the preliminary nature of our pilot feasibility study, there are several limitations of this work that need to be considered. First, these data were collected on a very small sample size of children with ASD without a comparison to typically developing children. Thus, the potential of this motion capture approach to understanding motor features that distinguish between ASD and typically developing children remains to be determined. Second, we focused our study on high functioning children and adolescents with ASD, so the potential utility of the GaitWayXR™ motor intervention might be limited in younger children with lower IQ, those who are minimally verbal or with clinically significant anxiety [43]. Third, the focus on a specific whole-body dynamic movement (jumping jack) was a limitation in understanding the potential to extend this low-cost motion capture approach to other types of movement tasks that rely more on visual feedback. For future work, it might be fruitful to develop other movement tasks that involve stringing together rapid sequences of simple movements using visual feedback to interact with objects in 3D space, given the significant impairments in using sensory feedback to control movement in ASD [44, 45]. This approach could model the kinematic properties of reaching movements including acceleration, deceleration and smoothness of movement in response to changes in visual conditions while initiating specific action chains in a virtual environment. Finally, related to this is the absence of haptic feedback during active gameplay that may pose a barrier to implementation of this VR intervention to improve motor skills in ASD, as there is significant evidence that autism is associated with difficulty using visual information and over-reliance on proprioception during motor learning [4, 44, 45]. Notwithstanding these limitations, we see our work as a precursor to more ecological technologies such as augmented reality which are already on the horizon to be widely available (e.g. Magic Leap 1). In anticipation for such technologies, we showed that it is possible to develop extended reality-based games in closed-loop with low-cost motion capture to track individuals’ movement patterns in real time and adapt the intervention accordingly.

## Conclusion

We built a prototype of a closed-loop virtual reality-based motor intervention which utilizes low-cost motion capture to adjust the gameplay in real time in youth with ASD. Our work was motivated to fill the gap in digital therapies that adopt widely available motion capture technology, VR technology and high-throughput data analytics for real-time adjustments during personalized interventions. Although the conclusions that can be drawn about the feasibility of VR-based motor training will need to be tempered based on the preliminary nature of this study, our study suggests that this novel approach is feasible in categorization of movement data into efficiency, synchrony and symmetry measures extracted from a low-cost motion tracking method for feature-based estimation of gross motor proficiency in ASD. A significant challenge to more widespread use of consumer-based motion capture will likely be a lack of automated methods for extracting and processing large datasets of therapeutic movements and removing extraneous noise during VR gaming interventions. Further development of these statistical machine learning methods may offer the potential for data-driven insights into therapy-induced motor improvements for personalization of treatment approaches in ASD and other neurodevelopmental disorders.

## Data Availability

The clinical datasets generated and/or analysed during the current study are available from the corresponding author upon request.
