# Correction to: Feasibility of a virtual reality-based exercise intervention and low-cost motion tracking method for estimation of motor proficiency in youth with autism spectrum disorder

**DOI:** 10.1186/s12984-022-01039-x

**Published:** 2022-06-24

**Authors:** Darren R. Hocking, Adel Ardalan, Hisham M. Abu-Rayya, Hassan Farhat, Anna Andoni, Rhoshel Lenroot, Stan Kachnowski

**Affiliations:** 1 Developmental Neuromotor and Cognition Lab, School of Psychology and Public Health, La Trobe University, Melbourne, VIC Australia; 2 Zuckerman Mind Brain Behavior Institute, Columbia University, New York, NY USA; 3 School of Social Sciences and Humanities, Doha Institute for Graduate Studies, Doha, Qatar; 4 School of Psychology and Public Health, La Trobe University, Melbourne, VIC Australia; 5 HITLAB, Healthcare Innovation & Technology Lab, Columbia University, New York, NY USA; 6 Department of Psychiatry, University of New Mexico, Albuquerque, NM USA

## Correction to: Journal of NeuroEngineering and Rehabilitation (2022) 19:1 https://doi.org/10.1186/s12984-021-00978-1

Following publication of the original article [1], the affiliation of the author “Hisham M. Abu‑Rayya” was incorrectly published as “School of Social Sciences and Humanities, Doha Institute for Graduate Studies, Doha, Qatar and School of Psychology and Public Health, La Trobe University, Melbourne, VIC, Australia.” The correct Affiliation is “School of Social Work, University of Haifa, Haifa, Israel and School of Psychology and Public Health, La Trobe University, Melbourne, Australia.”

The original article has been corrected.

## References

[1] Hocking DR, Ardalan A, Abu-Rayya HM, Farhat H, Andoni A, Lenroot R, Kachnowski S (2022). Feasibility of a virtual reality-based exercise intervention and low-cost motion tracking method for estimation of motor proficiency in youth with autism spectrum disorder. J Neuroeng Rehabil.

